# Editorial: Copy Number Variation in Rare Disorders

**DOI:** 10.3389/fgene.2022.898059

**Published:** 2022-04-05

**Authors:** Katalin Komlósi, Attila Gyenesei, Judit Bene

**Affiliations:** ^1^ Institute of Human Genetics, Medical Center, Faculty of Medicine, University of Freiburg, Freiburg, Germany; ^2^ Bioinformatics Research Group, Genomics and Bioinformatics Core Facility, Szentagothai Research Center, University of Pécs, Pécs, Hungary; ^3^ Department of Medical Genetics, Clinical Centre, Medical School, University of Pécs, Pécs, Hungary

**Keywords:** copy number variation (CNV), rare disorders, genomic disorders, Mendelian disease, genomic rearrangement

Copy number variation (CNV), encompassing losses or gains of relatively large genomic DNA segments, is one of the major sources of genetic diversity in humans (Zhang et al., 2009). Recent studies revealed that *de novo* locus-specific mutation rates appear much higher for CNVs than for SNPs (Lupski, 2007; Turner et al., 2008). CNVs comprise approximately 12–16% of the human genome and 3 to 7 rare CNVs can be found in an average genome (Harel and Lupski, 2018). The frequency of a CNV shows strong anticorrelation with its size and its gene density (Itsara et al., 2009). Although several CNVs are presumably benign, the role of CNVs in the pathogenesis of various diseases has increasingly gained attention nowadays thanks to the multiple sophisticated molecular laboratory technologies capable detecting various CNVs. Benign CNVs are frequently small, intergenic, or comprise genes that can tolerate copy number changes. Pathogenic CNVs are significantly enriched for genes involved in development and genes with constrained evolutionary patterns of gene duplication and loss (Rice and McLysaght, 2017). At the early era of CNV detection large CNVs (>500 kb) appeared to be associated with genomic disorders only; however, it is now clear that CNVs can also be involved in susceptibilities to complex traits, and nowadays there is an emerging evidence that CNVs may cause Mendelian diseases or sporadic traits as well (Zhang et al., 2009; Harel and Lupski, 2018).

The disease-causing genomic rearrangements can be either recurrent or non-recurrent. Recombination-based as well as replication-based mechanisms have been proposed to be responsible for the formation of CNVs such as nonallelic homologous recombination (NAHR), non-homologous end-joining (NHEJ), L1-mediated retrotransposition or Fork Stalling and Template Switching (FoSTeS) (Kazazian and Moran, 1998; Lupski and Stankiewicz, 2005; Korbel et al., 2007). There is a variety of molecular mechanisms by which CNVs can lead to abnormal phenotypes, encompassing dosage sensitivity of a gene within the CNV, gene interruption or gene fusion at the breakpoint junctions, deletion of a regulatory element, or unmasking of a recessive allele or functional polymorphism (Lupski and Stankiewicz, 2005). Moreover, CNVs can affect noncoding regulatory elements such as promoters or enhancers as well (Harel and Lupski, 2018).

The goal of this Research Topic was to provide the cutting-edge knowledge of CNVs leading to the development of rare disorders. Rare diseases are conditions that affect less than 5 in 10,000 people. To date more than 7,000 entities exist and the numbers are continuously increasing. Today little is known about the genetic background of still a significant portion of rare diseases, therefore their diagnostics is challenging. Furthermore, patients with undiagnosed genetic diseases often face a diagnostic odyssey, which lasts for 8 years on average. CNVs were initially proposed to represent a significant contribution to rare disease formation; however, there is now evidence from a recent study that CNV should be responsible for the disease phenotype in approx. 10% of cases (Truty et al., 2019).

The following topics were considered in this special issue:

• novel CNVs detected in rare disorders
• state-of-the-art technology for detection or evaluation of CNVs
• functional or animal studies related to the functional validation of CNVs
• comparative studies revealing phenotype–genotype correlation
• mechanism of non-coding CNVs in rare disorders and genotype-phenotype correlation

For our special topic we received five case reports, twelve original research reports and one review article, from which one review article, nine original research reports and three case reports were accepted for publication.

Pócza et al. provided a comprehensive review of the landscape of germline structural variation (SV) types and the various methodologies capable detecting SVs mainly focusing on cancer predisposition genes.

Xiao et al. investigated the phenotype of pediatric patients with 17q12 duplication syndrome. They demonstrated first in the literature that annular pancreas can be observed in approx. 20% of this patient cohort. Moreover, among the 15 genes encompassed within the 17q12 recurrent duplication/deletion region they verified the role of the *HNF1B* gene in pancreatic development using zebrafish studies.

Song et al. studied the association between a common prenatal ultrasound soft marker, echogenic intracardiac focus (EIF), and chromosomal abnormalities in pregnancies. No correlation was found between the appearance of isolated EIFs in early or mid-trimester and an increased risk of fetal chromosomal abnormalities. However, the persistence of EIFs in late trimester showed an association with a higher risk of pathology-related CNVs and may indicate heart development defects after birth.

Czakó et al. investigated the rare duplication of the Xp11.23p11.22 region in female patients with intellectual disability, epilepsy and minor anomalies. Based on their phenotypic and molecular cytogenetic data they concluded that Xp11.23p11.22 duplication can result in a neurodevelopmental disorder in females. A comparison of the studied patients with others reported so far clearly demonstrates that in addition to the breakpoints of the duplication and the role of the genes involved a number of other factors influencing gene expression may affect the symptoms observed in females with the Xp11.23p11.22 duplication.

Zodanu et al. investigated 22q11.2 copy number variations in pediatric and adult patients with congenital heart disease (CHD). Their data further confirmed previous findings that demonstrated high phenotypic diversity in 22q11.2 CNV carriers. Their results highlight the necessity for large-scale genetic screening of CHD-patients and the importance of early genetic diagnosis in their clinical management.

Cai et al. presented a family with Birt-Hogg-Dubé syndrome and a novel intragenic deletion spanning exons 10–14 in the *FLCN* gene detected by targeted next generation sequencing as a rare cause of the disease. Moreover, they demonstrated that the precision and accuracy of the applied NGS approach is similar to that of the MLPA (multiplex ligation-dependent probe amplification) technique.

Büki et al. performed genotype-phenotype association analyses in mostly paediatric patients with type-1, type-2, and atypical *NF1* microdeletions. In this study three novel atypical deletions were identified. The authors established that MLPA is a feasible, cost-effective technique for the identification and the classification of the *NF1* microdeletions.

Nevado et al. performed a deep phenotyping and genetic characterization of a large patient cohort with 5p minus syndrome. Within this clinically heterogeneous syndrome, around 39% of the studied patients carried clinically significant additional genomic rearrangements, mainly a duplication in other chromosomes which may explain part of the broad clinical spectrum.

Piras et al. investigated the genomic architecture of the *CFH-CFHR* region and characterized CNVs in a large cohort of patients with C3 glomerulopathy (C3G) and immune complex-mediated membranoproliferative glomerulonephritis (IC-MPGN). They identified novel CNVs leading to structural variants in 5 C3G and 2 IC-MPGN patients.

Nevado et al. performed a thorough clinical and genetic characterization in a cohort of 210 patients with Phelan-McDermid syndrome (PMS). Multiple variant types were observed among patients, including a significant number of small deletions and *SHANK3* sequence variants. Furthermore, multiple types of rearrangements were detected among microdeletion cases, including post-zygotic mosaicism, ring chromosome 22, unbalanced translocations and additional rearrangements at 22q13 as well as other copy number variations in other chromosomes. Their findings suggest that *SHANK3* plays an important role in this syndrome, but is probably not uniquely responsible for all the features in the PMS patients.

Pan and Fu reported the clinical characteristics of a patient carrying a large 10p deletion involving the 10p15.3–10p13 region as a second reported case in the literature. The patient had facial dysmorphism, swallowing dysfunction, hypoparathyroidism, hypocalcemia and neurological abnormalities.

Chen et al. reported two siblings with familial Beckwith-Wiedemann syndrome (BWS) due to a maternal deletion in *H19* and its upstream regulatory elements which can result in loss of function of the IGF2-H19 imprinting control element in the offspring and lead to BWS. Since studies on adult BWS patients are scarce the case report gives some insight on the presentation of BWS in adulthood and some of the potential reproductive issues such as subfertility or infertility in males.

Nagy et al. reported a family with distinct severity of expressive speech disorder, mild behavioral abnormality and dysmorphic features carrying a 7.87 Mb interstitial deletion of the 7q31.1q31.31 region involving the *FOXP2* gene. They concluded that the “phenotype first” then targeted diagnostic strategy can improve the diagnostic yield of speech disorders in the routine clinical practice.

In the 13 accepted manuscripts the contribution of CNVs to disease mechanism was investigated in a great variety of rare and ultra-rare diseases. 7/13 studies contributed to new clinical and genetic insights into rare microdeletion/microduplication syndromes while in 5/13 contributions the role of CNVs in disease mechanism was addressed in classically monogenic diseases.
